# Network mirroring for drug repositioning

**DOI:** 10.1186/s12911-017-0449-x

**Published:** 2017-05-18

**Authors:** Sunghong Park, Dong-gi Lee, Hyunjung Shin

**Affiliations:** Department of Industrial Engineering, Ajou University, 206 Worldcup-ro, Yeongtong-gu, Suwon, 16499 South Korea

**Keywords:** Drug repositioning, Disease network, Kullback-Leibler Divergence, Semi-Supervised Learning

## Abstract

**Background:**

Although drug discoveries can provide meaningful insights and significant enhancements in pharmaceutical field, the longevity and cost that it takes can be extensive where the success rate is low. In order to circumvent the problem, there has been increased interest in ‘Drug Repositioning’ where one searches for already approved drugs that have high potential of efficacy when applied to other diseases. To increase the success rate for drug repositioning, one considers stepwise screening and experiments based on biological reactions. Given the amount of drugs and diseases, however, the one-by-one procedure may be time consuming and expensive.

**Methods:**

In this study, we propose a machine learning based approach for efficiently selecting candidate diseases and drugs. We assume that if two diseases are similar, then a drug for one disease can be effective against the other disease too. For the procedure, we first construct two disease networks; one with disease-protein association and the other with disease-drug information. If two networks are dissimilar, in a sense that the edge distribution of a disease node differ, it indicates high potential for repositioning new candidate drugs for that disease. The Kullback-Leibler divergence is employed to measure difference of connections in two constructed disease networks. Lastly, we perform repositioning of drugs to the top 20% ranked diseases.

**Results:**

The results showed that F-measure of the proposed method was 0.75, outperforming 0.5 of greedy searching for the entire diseases. For the utility of the proposed method, it was applied to dementia and verified 75% accuracy for repositioned drugs assuming that there are not any known drugs to be used for dementia.

**Conclusion:**

This research has novelty in that it discovers drugs with high potential of repositioning based on disease networks with the quantitative measure. Through the study, it is expected to produce profound insights for possibility of undiscovered drug repositioning.

**Electronic supplementary material:**

The online version of this article (doi:10.1186/s12911-017-0449-x) contains supplementary material, which is available to authorized users.

## Background

Development of new drugs is carried out when there are no drugs to cure diseases or alleviate their clinical symptoms, or there are some motivations related to side effects [1]. Most of new drugs, which have been developed until now, used a method of de novo drug designing, which undergoes many phases covering from drug target discovery and screening to Absorption, Distribution, Metabolism, Excretion and Toxicity (ADMET) and Lead Optimization. Finally this method performs 3 phases of clinical tests in clinical areas and then approve a drug and commercializes it [2]. The whole processes for de novo drug discovery requires 10 ~ 17 years of period and tremendous cost of 300 ~ 600 million dollars, which is a deteriorated figure compared to 10 million dollar in 1970 and 100 million dollars in 2000 [3].

In order to solve problems of high cost and rate of failure with traditional drug discovery, drug repositioning has appeared [4]. Drug repositioning is a process to find probabilities that an already-approved drug could be applied to other diseases. This method, unlike conventional de novo method, has a most significant benefit that it could reduce required time to 3 ~ 12 years through in vitro or in vivo method [5]. Some of major success cases include a case that sildenafil is applied to erectile dysfunction and also a case that thalidomide is applied to multiple myeloma [6, 7]. This approach, however, has weaknesses in that it still relies on prior knowledge for manual method and clinical trials in wet bench and in that success stories are serendipitous and rare. Therefore, in silico drug repositioning which selects and predict new targets for drugs via computational approach are attracting people’s attentions [8]. In silico drug repositioning uses data for drugs, diseases and other relevant information. With such data, it performs a process to calculate probability of success for new indications found in conventional drugs by designing systematic algorithm and then finally predicts drug repositioning for selected high potential and evaluates its performance with accuracy [9]. So far, there have been numerous studies for in silico drug repositioning which could be divided into two mainstreams, drug-based approach and disease-based approach.

Drug-based approach attempts drug repositioning focusing on characteristics of drugs in terms of pharmaceutical aspects. Most of conventional researches predicted new targets of drugs by calculating similarities using drug-related information. Lamb et al. (2006) used molecule movement information for chemicals that are components of drugs [10], Keiser et al. (2009) took advantage of chemical structure and targeted protein information of drugs [11] while Chang et al. (2010) used tissue localization and gene expression pattern together [12]. However, information for drug’s chemical structure and characteristics contains numerous errors and moreover it’s hard to access such information due to ownership of drug manufacturers. Moreover, there is limitation for correct prediction due to complicated metabolic and pharmacokinetic transformations inside human body. Disease-based approach is started by identifying features of diseases at their gene or protein levels in terms of pathological aspects with proper medicine. In conventional studies, *Chiang* et al. (2009) approached a drug repositioning through “guilt by association” under assumption that if two diseases share few number of similar therapies, then a drug used for a certain disease could be used for other disease [13], *Campillos* et al. (2008) predicted new targets for drugs by calculating similarities between diseases based on side effect that appears from injection of drug [14]. However there are also limitations that lots of complex factors affect pathology of diseases and information for side effect should be well arranged and its amount should be also enough.

Although In silico drug repositioning methods are classified into two major ones, they mostly rely on an assumption based on similarity. Such assumption in drug-based approach is that similar drugs would have similar therapeutic influence upon the targets while assumption in disease-based approach is that similar diseases require similar therapy and thus the same drugs. Computational method to advance these assumptions is network-based modeling [15]. Drug repositioning based on network-based modeling is able to consider overall relations between diseases in terms of direct and indirect relations. In addition, this is able to extend relation between drugs and targets to “many-to-many” from “one-by-one” in terms of network [16]. Under these conditions, Suthram et al. (2010) attempted drug repositioning by structuring functional module network using molecular biological information and protein-protein interaction(PPI) [17].

**Fig. 1:**
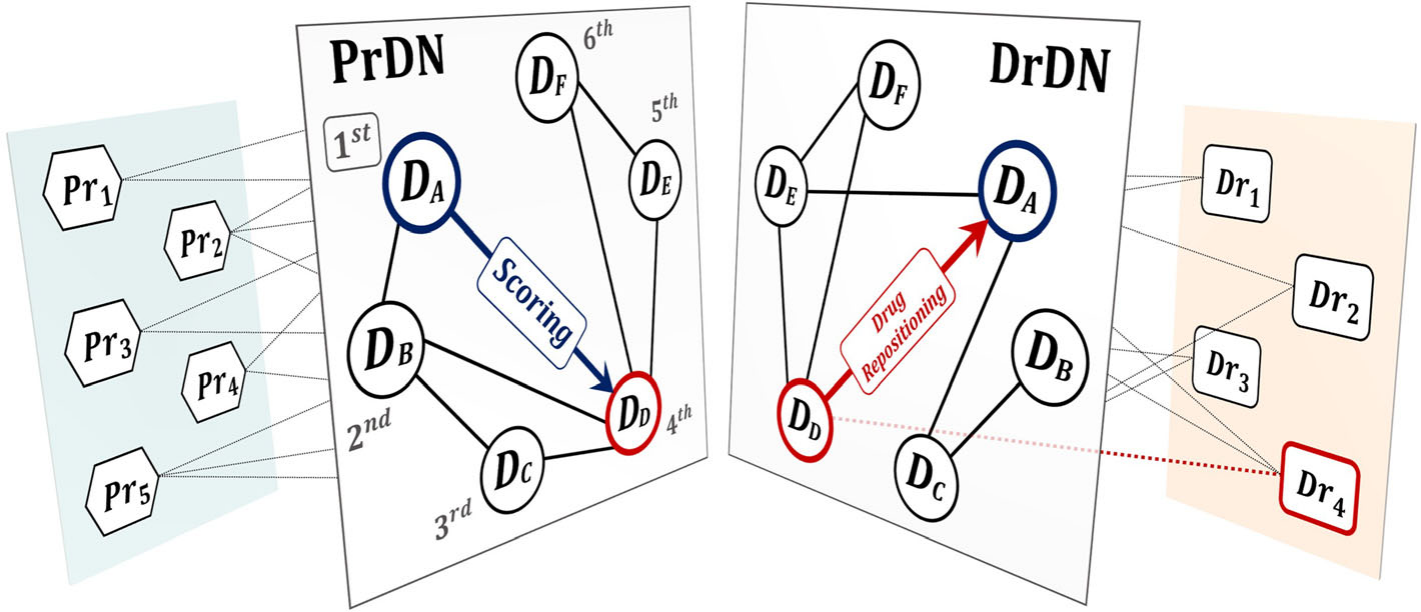
Network Mirroring. PrDN and DrDN are disease networks using protein information and drug information respectively. If we reflect the two networks, it would be easier to identify diseases with different connections. Different connections of disease nodes in two networks indicate that diseases which are similar in PrDN i.e. they share same protein information actually have different drug profiles. Given that diseases with similar bio-molecular characteristics can be treated by similar drugs, there is possibility of drug repositioning between these diseases

In this paper, we propose a methodology to implement drug repositioning via in silico to maximize effectiveness in terms of time and cost. From disease-based approach which is easy to be used with relatively lots of data, the proposed method includes network modeling which is easy to address relations between diseases and machine learning algorithm based on such relations. The proposed method is devised under an assumption that similar disease could be treated by similar drugs. If a disease with similar symptom doesn’t use similar drug even if two diseases are similar, then there could be an opportunity to reposition drugs between the two diseases. The proposed method is called *Network Mirroring* and its overview is shown in Fig. 1. Figure 1 shows a toy example in which it proposes 4 drugs (*Dr*_1_ ~ *Dr*_4_) with 5 proteins (*Pr*_1_ ~ *Pr*_5_) against 6 diseases (*D*_*A*_ ~ *D*_*F*_). Protein-based Disease Network(PrDN) and Drug-based Disease Network(DrDN) are disease networks constructed with protein and drug information, respectively. DrDN is reflected from PrDN through network mirroring and the relationships between disease nodes are identified. In the figure disease nodes are prioritized on the basis of difference in edges between diseases. From all six diseases, *D*_*A*_ is selected by first priority. For other five diseases, we applied a machine learning algorithm (Additional file 1) on PrDN to obtain scores. The most highly scored disease is believed to be most similar in terms of molecular biology. *D*_*D*_ is selected as the most similar disease compared to *D*_*A*_. Then, with identifying *Dr*_4_ to be used for *D*_*D*_ from disease-drug association and then repositions it to *D*_*A*_.

This paper consists of following sections: Section 2 explains procedures for Network Mirroring and Section 3 includes results of experiment that applied Network Mirroring to actual diseases. Section 4 represents our conclusion.

## Methods

### Network mirroring for drug repositioning

**Fig. 2:**
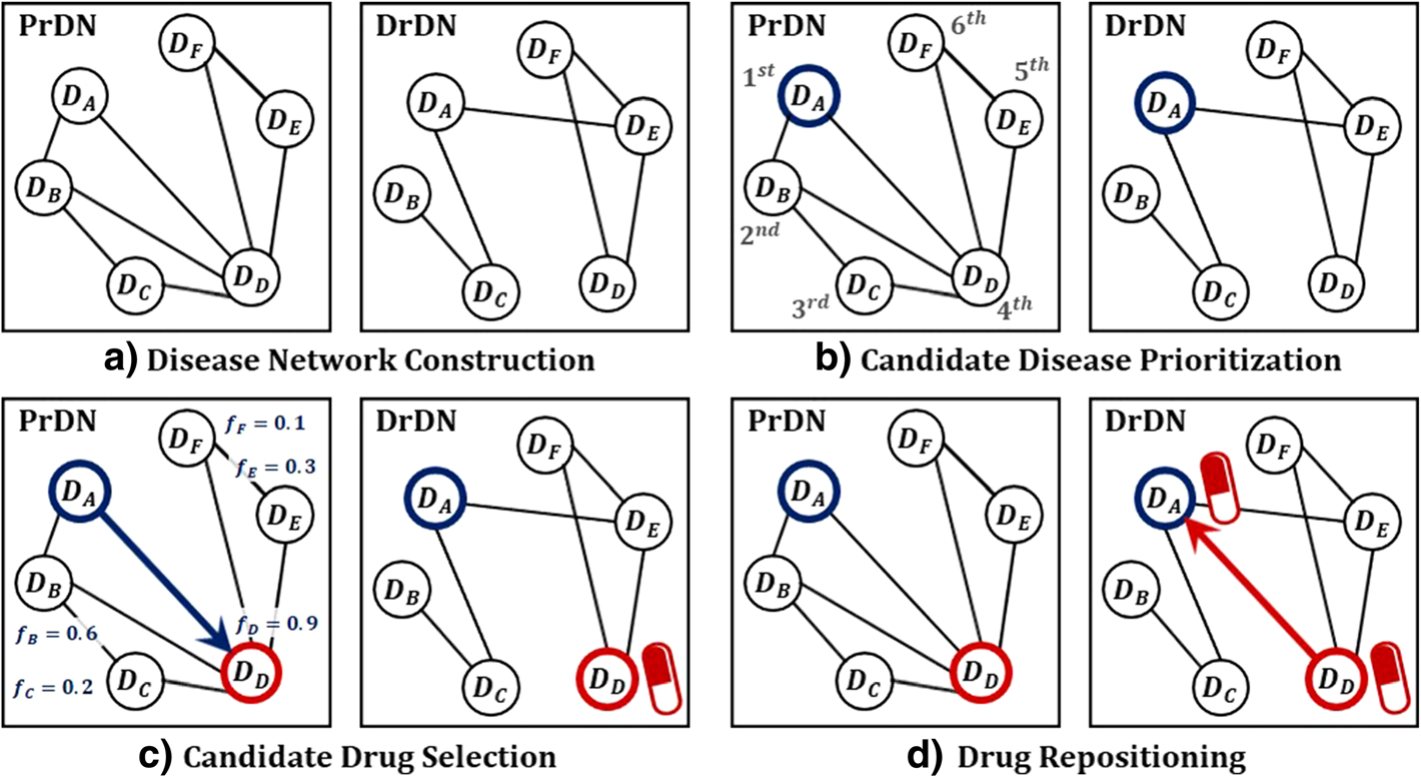
Schematic description of the proposed method. The proposed method consists of a total 4 steps: **a** it builds two disease networks PrDN and DrDN using protein and drug information respectively. **b** this step selects candidate disease by prioritizing diseases whose difference in edges is very high by mirroring DrDN from PrDN **c** it scores on other diseases against candidate disease through machine learning algorithm and then selects diseases whose score is high as similar diseases and then assigns candidate drugs which is used for such diseases **d** lastly, it repositions candidate drugs onto candidate disease

In this paper, we propose Network Mirroring as a new method to reposition drug. The proposed method is based on disease network. Disease network expresses relations between diseases by nodes and edges in graph in *G* = (*D*, *W*). Node set *D* is a disease and edge set *W* is calculated by similarity between diseases. In this case, meaning of similarity is varying depending on information used by calculating edges. Two disease networks are constructed by using different information. First one is a disease network based on protein information that diseases share and the other uses drug-related information for diseases. From the constructed networks, we can compare two disease networks. If drugs are well developed relying on molecular biological similarity between diseases, the two disease networks would be similar. However, such networks are different, there could be a possibility for drug repositioning. It is because diseases with similar molecular biology are likely to use same drugs. Network Mirroring based on such intuition consists of 4 steps. First, it builds two disease networks using protein and drug information respectively. Second, candidate disease is selected based on most different edges in two disease networks. Third, similar diseases are selected by similarity of candidate disease through machine learning algorithm and then candidate drugs are selected to be used for such diseases. Lastly, it repositions candidate drugs onto candidate disease. Schematic description for the proposed method is shown in Fig. 2.

### Disease network construction

From preceding studies on how to build disease network, Hidalgo et al. (2009) constructed network indicating co-occurrence between diseases by calculating edges based on records of patients [18]. Besides this, there are other studies constructing disease networks with various disease-related information such as genetic character, phenotype, protein interaction or metabolic pathway [19–23]. In this paper, we use tripartite information for protein-disease-drug to construct disease networks. This tripartite relation indicates a certain procedure for outbreak and treatment of diseases. It is because a disease is generated by abnormal protein and is treated by drug which targets such protein. Under this environment, we construct Protein-based Disease Network(PrDN) and Drug-based Disease Network(DrDN) by separating the tripartite information. Diseases on PrDN are connected to each other related to same protein [24–28]. In this case, connection between diseases indicate similarity of molecular biology [29]. Since the possibility of similar diseases being targeted by same drugs is high, PrDN indicates the potential of using same drugs for similar diseases. On the other hand, diseases on DrDN are connected with the number of shared drugs which are used for actual diseases [26, 28, 30]. Therefore, DrDN indicates status quo of using same drugs for similar diseases.

Disease networks are graphs, *PrDN* = (*D*, *W* ^*Pr*^) and *DrDN* = (*D*, *W* ^*Dr*^), that indicate connection between diseases with nodes and edges. Because two networks have same number and types of diseases, their node set is same but their edge set is different. Edges between diseases are calculated by Tanimoto similarity between vectors, which represent information of diseases [31, 32]. Tanimoto similarity, if its data type is binary or integer and if it’s sparse, is useful similarity measurement. Edge set *W* ^*Pr*^ uses protein vector while that of *W* ^*Dr*^ uses drug vector. Protein and drug vectors exist for each disease, and all vector elements are binary type. The weight value of each edge increases as the number of shared proteins or drugs between the two diseases increases. Equation (1) indicates calculation for similarity *w*_*ij*_ between Disease *i* and Disease *j*. **D**_**i**_ and **D**_**j**_ are vector for each disease while *D*_*ik*_ and *D*_*jk*_ is k^th^ component for protein or drug vector respectively.

1 $$ {w}_{ij}=\frac{{\displaystyle {\sum}_k{D}_{ik}}\cdot {D}_{jk}}{{\displaystyle {\sum}_k{D}_{ik}}+{\displaystyle {\sum}_k{D}_{jk}-{\displaystyle {\sum}_k{D}_{ik}}\cdot {D}_{jk}}} $$

### Candidate disease prioritization

In the candidate disease prioritization step, we select a disease for drug repositioning. For this purpose, the process searches diseases whose edge distribution is different by comparing PrDN and DrDN and then prioritizes them. Therefore, we apply the Kullback-Leibler(KL) divergence to compare all diseases quantitatively. The KL divergence is used to look into difference between two probability distributions [33–35]. The formula of KL divergence is shown in Eq. (2).

2 $$ K L\left( P\left|\right| Q\right)={\displaystyle \sum_i^N{P}_i\; ln\frac{P_i}{Q_i}} $$

where *P*_*i*_ and *Q*_*i*_ indicates probability function for probability variable *i*.

*KL*(*P* ∥ *Q*) indicates difference between a probability distribution *P* and *Q* (Note that the value is not symmetric if applied in reverse order, *Q* from *P*). KL is 0 if distribution of *P* and *Q* is same, otherwise it is other value than 0.

The proposed method in this study considers reflection of PrDN on DrDN since PrDN is a network providing information on potential drug repositioning. Therefore a probability distribution *P* in Eq. (2) is substituted by PrDN whereas a probability distribution *Q* is substituted by DrDN. However, KL divergence is calculated through probability value, pre-processing is required to convert *w*_*ij*_ into probability. In this case, edge is converted into exponential type to improve sparseness of data and then probability is calculated as shown in Eq. (3).

3 $$ {p}_{ij}=\frac{e^{w_{ij}}}{{\displaystyle {\sum}_k^N}{e}^{w_{ik}}} $$

where *N* denotes the number of diseases.

*p*_*ij*_ could be an expression of probability for weight of *D*_*j*_ among diseases connected to *D*_*i*_. Likewise, *q*_*ij*_ is also calculated by same equation. KL divergence is calculated for each disease and the bigger value is more highly prioritized by its orders. In other words, calculation of KL divergence for *i* ^th^ disease is expressed by Eq. (4).

4 $$ K{L}_i\left({p}_{i j}\parallel {q}_{i j}\right) = {\displaystyle \sum_i^N}{p}_{i j} \ln \frac{p_{i j}}{q_{i j}},\kern0.5em {p}_{i j},\ {q}_{i j}\in {R}^N $$

where *p*_*ij*_ and *q*_*ij*_ indicates probability value where *i* ^th^ disease is converted by PrDN and DrDN respectively.

**Fig. 3:**
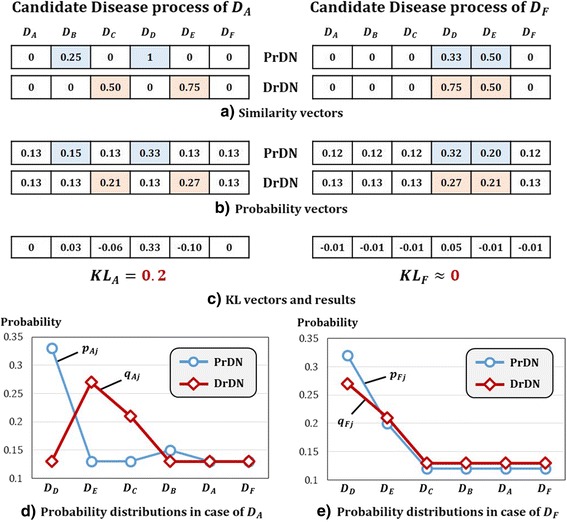
Toy example of Candidate Disease Prioritization. By comparing *D*_*A*_ and *D*_*F*_, figures show results of candidate disease prioritization by step-by-step. **a** expresses similarity vector for edges where *D*_*A*_ and *D*_*F*_ are connected to other diseases in PrDN and DrDN. **b** is a probability of similarity from **a** through pre-processing. **c** is KL value that is calculated between two diseases according to formula. *KL*_*A*_ is 0.2 and bigger than *KL*_*F*_ that is near 0. Therefore, intuitive decision for priority is digitized, we can see that same results are appearing. **d** and **e** are graphs which express probability distributions for two diseases in **b**. These graphs display such distribution by order of bigger values. The reason for big difference in KL value is evident by comparing **d** and **e**

With this process, upper σ% of diseases will be assigned to candidate disease for drug repositioning. σ is a user-specific parameter. We can see the example for candidate disease prioritization step through Fig. 2b. *D*_*A*_ is connected to *D*_*B*_ and *D*_*D*_ in PrDN while it is connected to *D*_*C*_ and *D*_*E*_ in DrDN, which means it is connected to totally different diseases between two disease networks. On the contrary, *D*_*F*_ is connected to *D*_*D*_ and *D*_*E*_ in both PrDN and DrDN. From assumption suggested by the proposed method, we can see intuitively that *D*_*A*_ with totally different connection is more likely to have probability of drug repositioning than *D*_*F*_ with perfectly same connection on two disease networks. This process and quantitative comparison procedures are shown in Fig. 3.

### Candidate drug selection and drug repositioning

Candidate drug selection step is a process to select drug to be repositioned for candidate disease. We define Candidate Drugs as drugs that are used for disease that are similar to candidate disease. Similar diseases are selected in a way that scores relations between candidate disease and other diseases on PrDN using machine learning algorithm and then the process selects disease whose score is bigger. For such scoring, graph-based Semi-Supervised Learning(SSL) algorithm is used [21]. SSL algorithm shows good performance especially when the number of labeled data is scarce compared to lots of data such as biomolecular and drug data. Among them, a suitable thing for network structure is graph-based SSL algorithm. When a graph and labels are given, SSL algorithm calculates predictive output, *f*-scores, for unlabeled nodes. See Appendix A. The bigger strength of connections between nodes leads to higher *f*-scores. The fact that higher *f*-scores for unlabeled nodes indicate that it is more similar to labeled nodes [22, 36, 37].

To assign similar diseases which are highly similar with candidate disease biologically, PrDN’s edge set *W* ^*Pr*^ is applied to the algorithm. A candidate disease node is set to be label ‘1’ and others are set to be ‘0’. Also, δ % of all diseases are selected to similar disease. δ is a user-specific parameter. Finally, all of drugs that used for similar diseases are chosen as candidate drugs for a candidate disease. This procedure is formulated as shown in Eq. (5).

5 $$ D{r}_i^C={\displaystyle \underset{j=1}{\overset{n_s}{\cup }} Drug\left({D}_j\right)} $$

where *n*_*s*_ = |{*S*(*D*_*i*_)}|, *D*_*j*_ ∈ *S*(*D*_*i*_), *D*_*i*_, *D*_*j*_ ∈ *PrDN*.

In (5), S(⋅) is a neighborhood function, which means *D*_*j*_ is one of similar diseases of *D*_*i*_. *Drug*(*D*_*j*_) means drugs used for disease *j* and *Dr*_*i*_^*C*^ means candidate drugs of disease *i*.

**Fig. 4:**
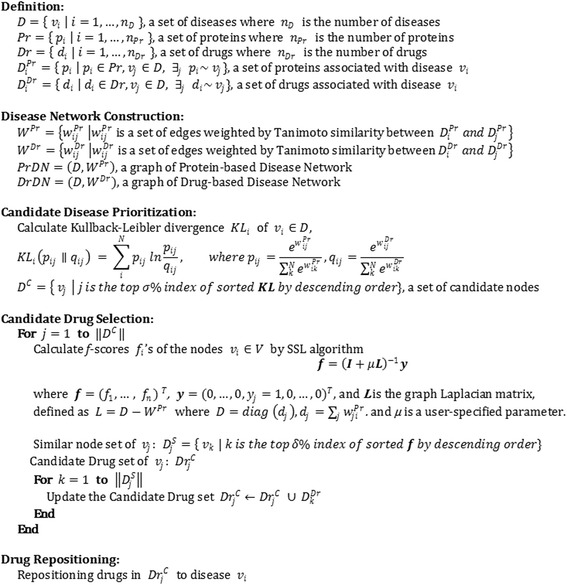
Pseudo Code of Network Mirroring

Toy example for candidate drug selection step is shown in Fig. 2c. *D*_*A*_is selected as candidate disease through the previous step. Therefore, label setting for all nodes is set to be {*D*_*A*_, *D*_*B*_, *D*_*C*_, *D*_*D*_, *D*_*E*_, *D*_*F*_} = {1, 0, 0, 0, 0, 0}. As the results of performing algorithm by applying PrDN’s edge set *W* ^*Pr*^ it’s proved that *f*-score for {*D*_*B*_, *D*_*C*_, *D*_*D*_, *D*_*E*_, *D*_*F*_} excepting *D*_*A*_is {0.6,0.2,0.9,0.3,0.1} respectively. Since it takes upper 20% (δ = 20) of such diseases, *D*_*D*_is finally selected. Consequently, drugs used for *D*_*D*_ are selected as candidate drugs. Finally, the last step of the process, drug repositioning by repositioning candidate drugs onto candidate disease. Figure 4 shows the pseudo code for Network Mirroring.

## Results and discussion

### Data

The proposed method is applied for all diseases which have association with proteins and drugs. We collected disease information from Medical Subject Headings(MeSH) in The National Library of Medicine(NLM) [38]. The relational information includes 161,223 disease-protein associations, 51,074 disease-drug associations and 91,450 drug-protein associations from multiple databases. With these information, we extracted diseases only having associations with protein and drug. Finally, we used 2890 diseases, 23,499 proteins and 4603 drugs information for PrDN and DrDN. We constructed PrDN using 161,223 disease-protein associations. When DrDN was constructed, we computed new disease-drug associations by combining existing disease-protein associations and drug-protein associations. In this case, disease and drug is related when they share same protein. The data used for construction of both networks are accessible in [39]. Table 1 summarizes sources and types of data used by the experiment.

**Table 1:** Data for diseases, proteins, drugs, and disease-protein associations, disease-drug associations, drug-protein associations

	Disease	Protein	Drug	Associations		
				Disease-Protein	Disease-Drug	Drug-Protein
Number of Data	2890	23,499	4603	161,223 relations	51,074 relations	91,450 relations
Sources	Medical subject Headings [38]	Entrez Gene [48]	PubChem [49]	GAD [24]	CTD [26]	PharmGKB [27]
				OMIM [25]	TTD [28]	T3DB [50]
				CTD [26]	DCDB [30]	DrugBank [51]
				PharmGKB [27]		ChEMBL [52]
				TTD [28]		CTD [26]
						TTD [28]
						DCDB [30]
						MATADOR [53]

### Results on validity of network mirroring

**Fig. 5:**
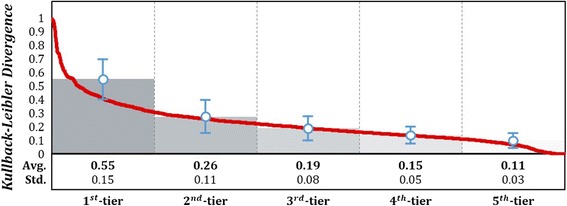
Kullback-Leibler divgergence value for entire diseases. The graph shows KL value on entire diseases by red line according to descending order. Average value of each tier is expressed by bar. By comparing PrDN and DrDN, diseases with different connection show higher KL value whereas ones with similar connection show lower KL value

We carried out verification as to how better performance drug repositioning shows when it is performed through the Network Mirroring. For this purpose, we divided all diseases into 5 tiers that is top 20% (σ = 20) unit depending on priority by candidate disease prioritization that is second step of Network Mirroring. Figure 5 indicates Kullback-Leibler divergence value for entire diseases and each tier.

For the next step, candidate drug selection and drug repositioning, we verified difference in performance for each tier. In this case, we compared with predicted result of drug repositioning with the reference experiment. In the reference experiment, we carried out greedy searching for the entire diseases. The experiment was repeated 10 times by 10-fold cross validation to disease-drug associations. The performance was measured on drug repositioning results in the last step of Network Mirroring. F-measure was used for performance measure. The process selects candidate drugs, which are all drugs used for similar diseases, and repositions them to candidate disease. Thus, the results consist of binary value (0 or 1). For binary results, F-measure is a suitable performance measurement method [40]. Eq. (6) is formula of F-measure.

6 $$ \boldsymbol{F}-\boldsymbol{measure}=\frac{2\left( precision\times recall\right)}{precision+ recall} $$

where $$ \boldsymbol{precision}=\frac{TP}{TP+ FP}\kern0.24em \boldsymbol{recall}=\frac{TP}{TP+ FN} $$

where TP, FP and FN indicate True Positive, False Positive, False Negative respectively in confusion matrix of Table 2.

**Table 2:** Confusion Matrix

		Predicted condition	
		Positive	Negative
Actual condition	Positive	True Positive	False Negative
	Negative	False Positive	True Negative

**Fig. 6:**
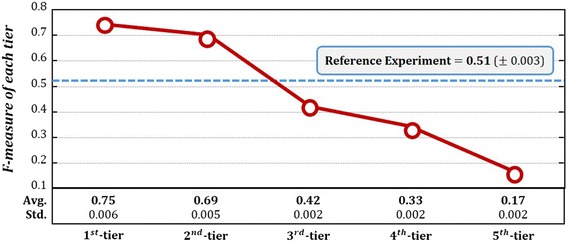
F-measure of each tier and entire diseases. The graph shows results of prediction for each tier through candidate drug selection and drug repositioning. The most precise tier has 1^***st***^-tier of KL value indicating 0.75 of F-measure performance. On the other hand, 5^***th***^-tier which falls on bottom 20% of KL value showed 0.17 that is the lowest accuracy. 5 tiers showed that they become more precise when their level is high. Reference experiment showed 0.51 performance. To summarize, the proposed method is believed to be a meaningful methodology to perform drug repositioning

Precision means the ratio of correct positive results to all positive results. Recall indicates the ratio of correct positive results to positive results that should have been returned. F-measure is a harmonic mean of them and Fig. 6 indicates F-measure for each tier.

### Results on utility of network mirroring

In this section, we show utility of Network Mirroring via dementia. The results are shown in step-by-step depending on the process concerning dementia. Dementia is caused by brain damage from various factors. If a normal person begins to suffer dementia, he or she shows critical disorder in cognitive skills. As their memory, language skills, decision making and abstractive thinking are deteriorated, it makes impossible to live a normal life [41, 42].

**Fig. 7:**
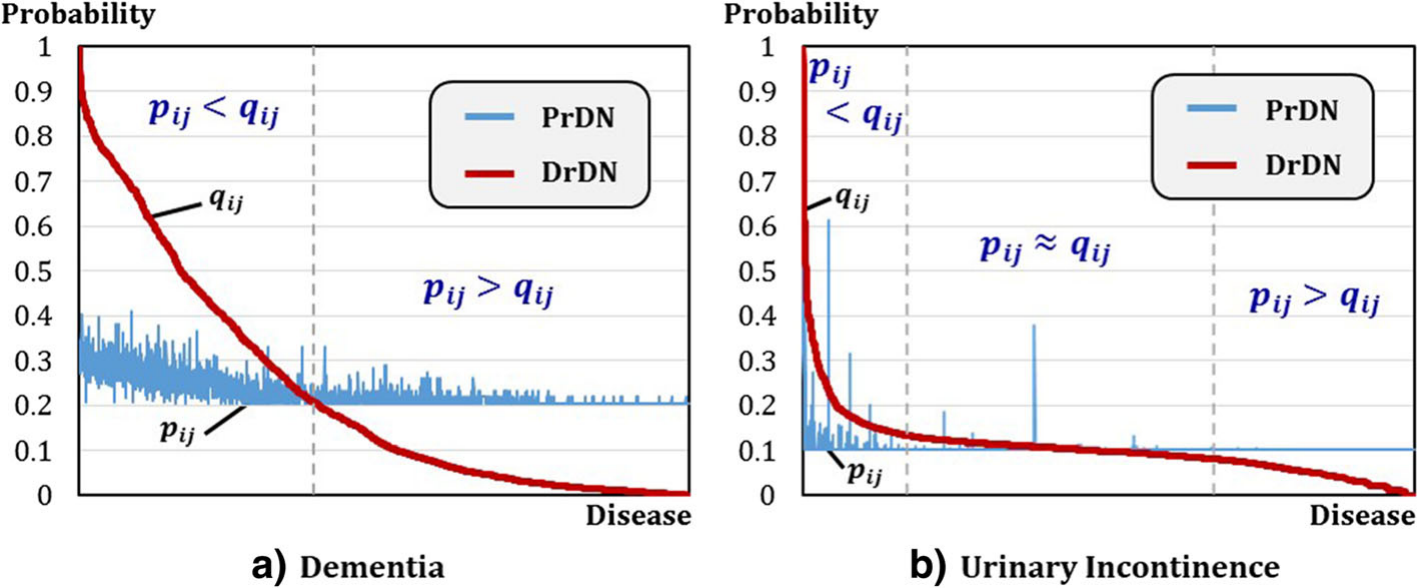
Probability Distributions in PrDN and DrDN for Dementia and Urinary Incontinence. The graphs show probability distributions in PrDN and DrDN to calculate KL value in the candidate disease prioritization step. **a** indicates a graph for dementia while **b** is a graph for urinary incontinence. Both graphs are lined-up by descending order of probability value. From the two graphs, **b** urinary incontinence shows a little bit of difference at both ends and almost overlapped interval is lengthy without significant difference. On the contrary, **a** dementia indicates significant difference without overlapped interval between PrDN and DrDN

First, we show results of the candidate disease prioritization step. Dementia, with its KL value of 0.68, belongs to upper 8% of entire diseases. For comparison, Urinary Incontinence, which falls on bottom 10% with 0.04 of KL value, is selected. Urinary incontinence is a disease that a person urinates unconsciously due to disorder in regulating bladder. It occurs along with overactive bladder, nocturia and other symptoms [43, 44]. Figure 7 shows probability distribution in PrDN and DrDN for dementia and urinary incontinence which shows big difference in KL value.

Next, we performed candidate drug selection and drug repositioning for dementia. Three similar diseases for dementia were selected from 2890 diseases, which is equivalent to 0.1% (δ = 0.1) of the entire disease. These three are lipid metabolism disorders, dyslipidemias and hypertriglyceridemia by the order of higher *f*-score. A total of 1296 candidate drugs are selected from similar diseases and they are all repositioned to dementia (Note that 1296 candidate drugs are ones targeting related proteins of three diseases). Dementia is related to 1300 drugs previously, 945 drugs out of 1296 repositioned ones covered existing drugs. Other 351 drugs are newly predicted drugs which are not identified yet. Actual effects on these are verified by clinical literature. Clinical literature showed the results of observing the progress of medication to patients in order to evaluate the effectiveness of medication. We used PubMed to search clinical information. As results of verification, 25 drugs out of newly repositioned 351 drugs for dementia are verified to be actually effective for dementia through clinical information literature. Proved results are shown with drugs and PMID in Table 3. To summarize, assuming that there are not any known drugs to be used for dementia, 970 drugs (945 + 25), 75% (970/1296) are verified to be repositioned via Network Mirroring.

**Table 3:** Validated Drugs via Literature Survey

Drug	PMID	Drug	PMID	Drug	PMID
Tolfenamic acid	25279694	Creatine	16434666	Vasopressins	21618004
Benzoic Acid	18317243	Putrescine	15324720	Diflunisal	25454121
Bupivacaine	25868211	Rivastigmine	27111084	Meclofenamic Acid	18164319
Hydrochlorothiazide	18604484	Indapamide	23743809	Tazarotene	22009441
Citrulline	25142005	Nabiloone	27232589	Lisnopril	25680080
Lutein	24200934	Azilsartan	25753301	Coenzyme A	24888381
Sucrose	26810632	Dronabinol	26271310	Inulin	27363809
Salicylic Acid	24113028	Quinapril	25680080	Fosinopril	26300914
Candesartan Cilexetil	12140732	25 drugs repositioned to Dementia are validated by clinical literatures.			

Now, we look into cases of Vasopressin, Tolfenamic acid and Creatine as major proved drugs through clinical literature. These three drugs, when they are repositioned for dementia, show high efficacy especially compared to other drugs.

#### Vasopressin

In several subtypes of frontotemporal dementia (FTD), damage to regions of the frontal and temporal lobes that occurs early in the disease course critically impairs emotional processing, social cognition, and behavior. Vasopressin can not only affect social cognition and behavior, but also serve as the potential implications for these agents as novel treatments in FTD [45].

#### Tolfenamic acid

Tolfenamic acid lowers the levels of tau, which forms pathological aggregates in Alzheimer’s disease and other tauopathies, by promoting the degradation of the transcription factor specificity protein 1 which regulates tau transcription [46].

#### Creatine

Sixty four participants were able to keep their condition healthy and stable by taking 8 g of creatine during 16 weeks of clinical trial. In addition, efficacy of creatine to treat dementia could be verified through Serum8-hydroxy-2'-deoxyguanosine (8OH2'dG) levels indicating oxidative injury to DNA. Although this value is rapidly increasing if condition for a patient aggravates, it could be reduced to a normal condition by creatine treatment. Therefore, if creatine is repositioned to dementia, it’s believed to be effective for treatment [47].

## Conclusion

In this paper, we propose Network Mirroring for drug repositioning. The proposed method starts from an assumption that diseases with similar molecular biological characteristics are likely to use same drugs. We constructed two disease networks, PrDN and DrDN from protein information and drug information and reflects them. To check whether or not diseases with similar molecular biological characteristics use similar drugs, the criterion is PrDN. If they are different, such condition could be regarded as remaining room for drug repositioning. We used Kullback-Leibler divergence for quantitative comparison. Through the process, we select candidate disease by prioritizing a list of diseases suitable for drug repositioning. Then, we determine similar diseases with the candidate disease based on graph-based SSL algorithm. From similar diseases, we select candidate drugs. Finally, we complete Network Mirroring for drug repositioning which repositions candidate drugs to candidate disease.

For verification of the proposed method, we applied it to 2890 diseases, 23,499 proteins and 4603 drugs information. From the results, the proposed method preferably repositions drugs in top 20% of diseases more effectively than accessing to entire diseases. To observe the utility of the proposed method, it was applied to dementia. The selected drugs with Network Mirroring coincides with existing drugs in usage. In addition, it also discovered drugs with high potential of repositioning and the drugs were verified through clinical literature. Through the study, It is expected to produce profound insights for possibility of undiscovered drug repositioning.

For future works, we can consider performance comparison with existing works for validation and develop Network Mirroring into more sophisticated algorithm. In the aspect of utility, by integrating various information related to diseases, we plan to complement PrDN and extend Network Mirroring not only to dementia but also to other various diseases. In addition, we plan to carry out more studies for discovering new repositioned drugs for candidate diseases by considering information regarding drug analogues used for treatment.

## Additional file

Additional file 1: Graph-based Semi-Supervised Learning. (DOCX 38 kb)

## References

[1] Hughes J, Rees S, Kalindjian S, Philpott K (2011). Principles of early drug discovery. Br J Pharmacol.

[2] Ashburn TT, Thor KB (2004). Drug repositioning: identifying and developing new uses for existing drugs. Nat Rev Drug Discov.

[3] Scannell JW, Blanckley A, Boldon H, Warrington B (2012). Diagnosing the decline in pharmaceutical R&D efficiency. Nat Rev Drug Discov.

[4] Khanna I (2012). Drug discovery in pharmaceutical industry: productivity challenges and trends. Drug Discov Today.

[5] Barratt MJ, Frail DE. Drug repositioning: Bringing new life to shelved assets and existing drugs. Wiley; 2012.

[6] Goldstein I, Lue TF, Padma-Nathan H, Rosen RC, Steers WD, Wicker PA (1998). Oral sildenafil in the treatment of erectile dysfunction. N Engl J Med.

[7] Singhal S, Mehta J, Desikan R, Ayers D, Roberson P, Eddlemon P, Munshi N, Anaissie E, Wilson C, Dhodapkar M (1999). Antitumor activity of thalidomide in refractory multiple myeloma. N Engl J Med.

[8] Dudley JT, Deshpande T, Butte AJ. Exploiting drug–disease relationships for computational drug repositioning. Brief Bioinform. 2011;12(4):303–11. 10.1093/bib/bbr013 PMC3137933 21690101

[9] Hurle M, Yang L, Xie Q, Rajpal D, Sanseau P, Agarwal P. Computational drug repositioning: from data to therapeutics. Clin Pharmacol Ther. 2013;93(4):335–41. 10.1038/clpt.2013.1 23443757

[10] Lamb J, Crawford ED, Peck D, Modell JW, Blat IC, Wrobel MJ, Lerner J, Brunet J-P, Subramanian A, Ross KN (2006). The Connectivity Map: using gene-expression signatures to connect small molecules, genes, and disease. Science.

[11] Keiser MJ, Setola V, Irwin JJ, Laggner C, Abbas AI, Hufeisen SJ, Jensen NH, Kuijer MB, Matos RC, Tran TB (2009). Predicting new molecular targets for known drugs. Nature.

[12] Chang RL, Xie L, Xie L, Bourne PE, Palsson BØ (2010). Drug off-target effects predicted using structural analysis in the context of a metabolic network model. PLoS Comput Biol.

[13] Chiang AP, Butte AJ (2009). Systematic evaluation of drug-disease relationships to identify leads for novel drug uses. Clin Pharmacol Ther.

[14] Campillos M, Kuhn M, Gavin A-C, Jensen LJ, Bork P (2008). Drug target identification using side-effect similarity. Science.

[15] Liu Z, Fang H, Reagan K, Xu X, Mendrick DL, Slikker W, Tong W (2013). In silico drug repositioning–what we need to know. Drug Discov Today.

[16] Yıldırım MA, Goh K-I, Cusick ME, Barabási A-L, Vidal M (2007). Drug—target network. Nat Biotechnol.

[17] Suthram S, Dudley JT, Chiang AP, Chen R, Hastie TJ, Butte AJ (2010). Network-based elucidation of human disease similarities reveals common functional modules enriched for pluripotent drug targets. PLoS Comput Biol.

[18] Hidalgo CA, Blumm N, Barabási A-L, Christakis NA (2009). A dynamic network approach for the study of human phenotypes. PLoS Comput Biol.

[19] Davis DA, Chawla NV (2011). Exploring and exploiting disease interactions from multi-relational gene and phenotype networks. PLoS One.

[20] Li Y, Agarwal P (2009). A pathway-based view of human diseases and disease relationships. PLoS One.

[21] Nam Y, Kim M, Lee K, Shin H. CLASH: Complementary linkage with anchoring and scoring for heterogeneous BioMolecular and clinical data. BMC Med inform decis making. 2016;16(Suppl 3):72. 10.1186/s12911-016-0315-2 PMC4959382 27454118

[22] Shin H, Nam Y, Lee D-g, Bang S (2014). The Translational Disease Network—from proetin Interatction to Disease Co-occurrence. Proc of 4th Translational Bioinformatics Conference (TBC) 2014 [US Patent-14/920447].

[23] Zhang X, Zhang R, Jiang Y, Sun P, Tang G, Wang X, Lv H, Li X (2011). The expanded human disease network combining protein–protein interaction information. Eur J Hum Genet.

[24] Becker KG, Barnes KC, Bright TJ, Wang SA (2004). The genetic association database. Nat Genet.

[25] Hamosh A, Scott AF, Amberger JS, Bocchini CA, McKusick VA (2005). Online Mendelian Inheritance in Man (OMIM), a knowledgebase of human genes and genetic disorders. Nucleic Acids Res.

[26] Davis AP, Murphy CG, Johnson R, Lay JM, Lennon-Hopkins K, Saraceni-Richards C, Sciaky D, King BL, Rosenstein MC, Wiegers TC (2013). The comparative toxicogenomics database: update 2013. Nucleic Acids Res.

[27] Whirl-Carrillo M, McDonagh E, Hebert J, Gong L, Sangkuhl K, Thorn C, Altman R, Klein TE (2012). Pharmacogenomics knowledge for personalized medicine. Clin Pharmacol Ther.

[28] Chen X, Ji ZL, Chen YZ (2002). TTD: therapeutic target database. Nucleic Acids Res.

[29] Alberts B, Bray D, Lewis J, Raff M, Roberts K, Watson JD, Grimstone A. Molecular Biology of the Cell (3rd edn). Trends Biochem Sci. 1995;20(5):210–0.

[30] Liu Y, Wei Q, Yu G, Gai W, Li Y, Chen X (2014). DCDB 2.0: a major update of the drug combination database. Database.

[31] Jaccard P. Distribution de la flore alpine dans le bassin des Dranses et dans quelques régions voisines. Bull. Soc. Vaud. Sci. Nat. 1901;37:241–72.

[32] Tanimoto TT (1957). IBM internal report.

[33] Kullback S. Information theory and statistics. Courier Corporation; 1997.

[34] Kullback S, Leibler RA (1951). On information and sufficiency. Ann Math Stat.

[35] Weinstein E, Feder M, Oppenheim AV (1990). Sequential algorithms for parameter estimation based on the Kullback-Leibler information measure. IEEE Trans Acoust Speech Signal Process.

[36] Chapelle O, Scholkopf B, Zien A (2006). Semi-supervised learning. 2006.

[37] Kim J, Shin H (2013). Breast cancer survivability prediction using labeled, unlabeled, and pseudo-labeled patient data. J Am Med Inform Assoc.

[38] Lipscomb CE (2000). Medical subject headings (MeSH). Bull Med Libr Assoc.

[39] PharmDB [http://pharmdb.org]. Accessed 11 Jan 2016.

[40] Powers DM (2011). Evaluation: from precision, recall and F-measure to ROC, informedness, markedness and correlation.

[41] Ferri CP, Prince M, Brayne C, Brodaty H, Fratiglioni L, Ganguli M, Hall K, Hasegawa K, Hendrie H, Huang Y (2006). Global prevalence of dementia: a Delphi consensus study. Lancet.

[42] Prince M, Bryce R, Albanese E, Wimo A, Ribeiro W, Ferri CP (2013). The global prevalence of dementia: a systematic review and metaanalysis. Alzheimers Dement.

[43] Irwin DE, Milsom I, Hunskaar S, Reilly K, Kopp Z, Herschorn S, Coyne K, Kelleher C, Hampel C, Artibani W (2006). Population-based survey of urinary incontinence, overactive bladder, and other lower urinary tract symptoms in five countries: results of the EPIC study. Eur Urol.

[44] Thomas TM, Plymat KR, Blannin J, Meade T (1980). Prevalence of urinary incontinence. Br Med J.

[45] Finger EC (2011). New potential therapeutic approaches in frontotemporal dementia: oxytocin, vasopressin, and social cognition. J Mol Neurosci.

[46] Adwan L, Subaiea GM, Basha R, Zawia NH (2015). Tolfenamic acid reduces tau and CDK5 levels: implications for dementia and tauopathies. J Neurochem.

[47] Hersch S, Gevorkian S, Marder K, Moskowitz C, Feigin A, Cox M, Como P, Zimmerman C, Lin M, Zhang L (2006). Creatine in Huntington disease is safe, tolerable, bioavailable in brain and reduces serum 8OH2′ dG. Neurology.

[48] Maglott D, Ostell J, Pruitt KD, Tatusova T (2005). Entrez Gene: gene-centered information at NCBI. Nucleic Acids Res.

[49] Wang Y, Suzek T, Zhang J, Wang J, He S, Cheng T, Shoemaker BA, Gindulyte A, Bryant SH. PubChem bioassay: 2014 update. Nucleic Acids Res. 2013;gkt978. 10.1093/nar/gkt978 PMC3965008 24198245

[50] Lim E, Pon A, Djoumbou Y, Knox C, Shrivastava S, Guo AC, Neveu V, Wishart DS (2010). T3DB: a comprehensively annotated database of common toxins and their targets. Nucleic Acids Res.

[51] Wishart DS, Knox C, Guo AC, Shrivastava S, Hassanali M, Stothard P, Chang Z, Woolsey J (2006). DrugBank: a comprehensive resource for in silico drug discovery and exploration. Nucleic Acids Res.

[52] Gaulton A, Bellis LJ, Bento AP, Chambers J, Davies M, Hersey A, Light Y, McGlinchey S, Michalovich D, Al-Lazikani B (2012). ChEMBL: a large-scale bioactivity database for drug discovery. Nucleic Acids Res.

[53] Günther S, Kuhn M, Dunkel M, Campillos M, Senger C, Petsalaki E, Ahmed J, Urdiales EG, Gewiess A, Jensen LJ (2008). SuperTarget and Matador: resources for exploring drug-target relationships. Nucleic Acids Res.

