# Supplementary material for: Network mirroring for drug repositioning
Source: BMC Med Inform Decis Mak. 2017 May 18;17(Suppl 1):55. doi: 10.1186/s12911-017-0449-x (PMC5444046; doi:10.1186/s12911-017-0449-x)
Supplement: Additional file 1: — Graph-based Semi-Supervised Learning. (DOCX 38 kb) [file 12911_2017_449_MOESM1_ESM.docx]

Additional file

**Additional file 1. Graph-based Semi-Supervised Learning**

Disease network is a graph that represents *disease-disease* association with nodes and edges. A node in a disease network represent disease and edges represent associations between nodes. Given a disease network, we can apply graph-based semi-supervised learning (SSL) algorithm to calculate the scores when a target disease is given. The target disease for our study is the candidate disease for drug repositioning. In order to apply the algorithm, we set labels for diseases. Candidate disease node is labeled with '1' and other diseases are '0' as unlabeled nodes. Based on these label settings, SSL scores unlabeled nodes and derives the *f*-score as a result. More details about the implementation of algorithm can be found in [21, 22, 36].

In graph-based SSL, a connected graph $G=\left( V,W \right)$ is constructed where the nodes $V$ represent the labeled and unlabeled data points while the edges $W$ reflect the similarity between data points. The value of the similarity is represented by a matrix $W=\{w_{ij}\}$ where $w_{ij}$ is the edge between nodes $v_{i}$ and $v_{j}$. In this study, Tanimoto method is used to calculate the edge.

If the numbers of total nodes, labeled nodes, and unlabeled nodes are $n$, $l$, and $u$ respectively, $n$ would be a sum of $l$ and $u$. Also, we can represent the node set as $V\boldsymbol{=}{(v}_{1},\ldots,v_{l},v_{l+1}, \ldots, v_{n=l+u})$ and the label setting of them as $\boldsymbol{y}={(y_{1}=1,\ldots,y_{l}=1,y_{l+1}=0, \ldots, y_{n=l+u}=0)}^{T}$. Then the label information are able to propagate from labeled node to unlabeled node, and finally we can derive $\boldsymbol{f}={(f_{1},\ldots,f_{l},f_{l+1}, \ldots, f_{n=l+u})}^{T}$ as the result of label propagation.

There are two conditions for optimizing ***f****.* One is a loss function which indicates that $f_{i}$ should be close to given label of $y_{i}$ in labeled nodes. Another is a label smoothness which indicates that $f_{i}$ should not be too different from $f_{i}$ for the neighboring nodes. To calculate  ***f***, we have to minimize the following quadratic function which including both conditions.

$$\min_{\boldsymbol{f}} \left( \boldsymbol{f}-\boldsymbol{y} \right)^{T}\left( \boldsymbol{f}-\boldsymbol{y} \right)+\mu\boldsymbol{f}^{T}\boldsymbol{Lf}$$

where ***L*** is the graph Laplacian matrix, defined as $L=D-W^{Pr}$ where $D=diag \left( d_{j} \right), d_{j}=\sum_{j} w_{ji}^{Pr}$*.* In this study, we give label only candidate diseases and applying SSL algorithm one by one, therefore, the label setting is transformed from $\boldsymbol{y}={(y_{1}, \ldots, y_{l}, 0, \ldots, 0)}^{T}$ to $\boldsymbol{y}={(0, \ldots, 0, y_{i}=1, 0, \ldots, 0)}^{T}$ when calculates $\boldsymbol{f}$ of $i^{th}$ candidate disease from total $l$ candidate diseases. *μ* is a user-specified parameter and it trades off loss and smoothness. Thus, the solution of this problem becomes

$\boldsymbol{f}=\left( \boldsymbol{I}+\mu\boldsymbol{L} \right)^{-1}\boldsymbol{y}$*.*
